# The Role of Stereotactic Radiotherapy in the Management of Melanoma, A Retrospective Single Institute Preliminary Study of 30 Patients

**DOI:** 10.3389/pore.2022.1610550

**Published:** 2022-09-08

**Authors:** Mihály Kispál, Levente Zsolt Jánváry, Tímea Balatoni, Stelczer Gábor, Imre Fedorcsák, Bőcs Katalin, István Kenessey, Gabriella Liszkay

**Affiliations:** ^1^ Department of Dermato-Oncology, National Institute of Oncology, Budapest, Hungary; ^2^ Department of Radiotherapy Centre, National Institute of Oncology, Budapest, Hungary; ^3^ National Institute of Mental Health, Neurology and Neurosurgery, Budapest, Hungary; ^4^ Department of Radiology, National Institute of Oncology, Budapest, Hungary; ^5^ National Cancer Registry, National Institute of Oncology, Budapest, Hungary

**Keywords:** skin cancer, stereotactic radiation therapy, targeted therapy, immunotherapy, melanoma

## Abstract

Cutaneous melanoma is the third most common type of skin cancer in the world. The incidence of melanoma is increasing in most countries, however, mortality seems to be slowly decreasing. The treatment of advanced cutaneous melanoma changed radically since 2011. The new therapeutic modalities, such as immuno- and targeted therapies give a chance to successfully reach more prolonged progression-free survival (PFS) and overall survival (OS) in patients with metastatic melanoma. Despite the great therapeutic benefit, most patients eventually develop resistance to these therapies, and the disease will progress. In some cases oligoprogression develops. In those cases local therapy, such as stereotactic radiotherapy can make it possible to continue the previously applied effective medical treatment for the benefit of patients. In our study of a total of 30 patients—20 of them received pre-treatment with systemic medical therapy—received stereotactic radiotherapy using various systems, in the National Institute of Oncology, Hungary, Budapest. We managed to prolong the systemic therapy for 12.5 months median period with the assistance of CyberKnife technique. Therapy related adverse events were mostly tolerable with only 3% of Grade 3 toxicity. We concluded that stereotactic radiotherapy and stereotactic radiosurgery, are safe, and effective therapeutic modalities for regional tumor control in cases of oligoprogression.

## Introduction

Cutaneous melanoma is the most lethal form of skin cancer. In 2020, 324,635 new melanoma cases were estimated worldwide (1). The incidence of melanoma is increasing in most countries: in the US, the number of new melanoma cases in 2021 exceeded those of 2020 by 5.8% (2). However, the mortality seems to be slowly decreasing, thanks to efficient secondary prevention, and to new therapeutic modalities since 2011 (3).

Cutaneous melanoma has a high potential to metastatize, brain metastases occur in 10%–40% of patients (4). The systemic therapy of advanced melanoma has been completely changed by having innovative therapies available (targeted BRAF-MEK inhibitors and immunotherapy with PD1/PD-L1 and CTLA-4 checkpoint inhibitors). We can reach rapid therapeutic effects with the use of targeted therapy and long-standing survival with checkpoint inhibitors (CPI) (5,6). In cases of minimal progression of the disease (progression is at a limited degree, meaning it affects less than three regions, and one or two metastases, while the systemic therapy controls the disease on all the other sites), when it is mostly controllable, the development of innovative technical tools of radiotherapy makes it possible to continue effective medical treatment. Synchronously given radiation and systemic therapy increases the incidence of serious adverse events. Side-effect profile is more favorable in simultaneous radiation therapy and immunotherapy (7). The aim of our retrospective single institute study was to (possessing innovative therapies) evaluate the effects and side-effects of stereotactic radiosurgery (SRS) and stereotactic radiotherapy (SRT).

## Materials and Methods

### Patients

Between 2018 January and 2020 October, from our target population 30 melanoma patients were eligible for this analysis, >18 years old, with oligometastases, or oligoprogression present, received stereotactic radiotherapy, in the National Institute of Oncology, Budapest, Hungary. Patients with a median follow up of 20 months were included in this study. Therapeutic choices and indication of stereotactic radiotherapy were always based on the decision of a multi-disciplinary board, which included a dermato-oncologist, histopathologist a radiation oncologist, a neurosurgeon, a radiologist, and a general surgeon. Systemic targeted, immuno-, and chemotherapy was permitted. At targeted therapies, the drug was suspended 1 day before the radiotherapy, and was continued 1 day after the treatment. In case of immunotherapies it was not necessary to suspend treatment.

### Targeted Radiotherapeutic Methods

The CyberKnife (Accuray Inc., Sunnyvale, CA, United States) is the state-of-the-art technology of radiotherapy, a compact linear accelerator with an integrated image guiding system. Usually, one session of radiotherapy with the CyberKnife system lasts 20–40 min long.

Apart from CyberKnife, the radiotherapeutic treatments can be performed with other new generation systems.

LINAC-based systems (Truebeam, Vitalbeam, Varian Medical Systems, Palo Alto, CA, United States): Currently in our Institute we are using Varian Truebeam and VitalBeam models to perform stereotactic body radiation therapy (SBRT). These systems are rotating in one plane around the patient and they can concentrate the radiation into the center of rotation. LINAC based SBRT has an average period of 8–12 min.

The most important radiotherapeutic indications in advanced melanoma are the metastases in the brain, lungs, liver and vertebral column. Besides, infrequently we can treat lesions in the abdominal or thoracic region and renal, adrenal gland. In our Institute currently the treatment method of brain metastases takes place with the CyberKnife system. One of the advantages being that instead of the previously used invasive and painful headframes in this treatment, we use personalized, thermoplastic frames, thanks to the integrated image guiding system, which can detect and correct even the smallest shift. We use primarily the CyberKnife in case of extracranial metastases. If the risks, or the complications of the gold marker implantation is too high (bleeding, perforation, pneumothorax), we prefer the use of traditional non-invasive LINAC based systems when we could profit from the 3D image-guiding system, which could correctly localize the tumor, however in this case it is difficult to follow the respiratory movements.

### Radiotherapy Fractionation

For brain metastases smaller than 3 cm one-fraction SRS treatment was performed with 1x18-20Gy. Brain metastasis of more than 3 cm in greatest diameter or lesions on the near proximity of brainstem or optical chiasma, were treated with fractionated irradiation schedules of 3x8-10Gy or 5x5-8Gy.

In peripheral lung metastases 3x18 and 5x12Gy were given, centrally localized lung metastases were irardiated with 8x7,5Gy.

Radiotherapy doses for hepatic metastases can be varied between 3x15-18 Gy, while abdominal lymph nodes, and spinal metastases were treated with 5x7-8Gy.

### Follow Up and Safety Assessments

The radiological follow-up consisted of a CT scan of the head, neck, chest, abdominal and pelvic region in patients without intracerebral dissemination at screening and every 3 months thereafter and a magnetic resonance imaging scan of the brain if this was an initial site of disease. CT and magnetic resonance (MRI) images were evaluated by Response Evaluation Criteria in Solid Tumours (RECIST) version 1.1 (8) and immuno RECIST (9).

Adverse events (AE) were recorded both during and after the systemic and the radiotherapy. AEs were graded using the Common Terminology Criteria for Adverse Events (CTCAE) version 5.0 (10).

### Statistical Analysis

Next to descriptive statistics PFS and OS were evaluated by using the Kaplan-Meier method and log-rank analysis. Survival periods were determined as the initiation of systematic treatment or radiotherapy in patients who did not received systemic therapy, to the date of last visit or defined complete event (death, progression) (PFS was defined as the time period to progression, other than oligoprogression). Differences were considered to be statistically significant when *p*-value proved be lower than 0.05. All statistical calculations were performed by Statistica 13.4 (TIBCO Software, Palo Alto, CA, United States).

## Results

### Patients Characteristics

Thirty patients were treated with stereotactic radiation between January 2018 and October 2020. Baseline characteristics are summarized in Table 1. 22 patients (74%) had brain metastases (M1D) at the start of the radiation treatment, while four patients (13%) had visceral metastases (M1C) and another four patients (13%) had metastases in the lungs (M1B). The base lactate dehydrogenase (LDH) blood levels were normal in 18 patients (60%), elevated in 10 patients (33%) and was unknown in two patients (7%).

**TABLE 1:** Patient’s characteristics.

Variables	No (%)
All patients	30 (100%)
Age (years)	
Median (range)	60 (26–75)
Gender	
Male	18 (60%)
Female	12 (40%)
ECOG performance status	
0	20 (66%)
1	8 (27%)
2	2 (7%)
M stage (AJCC 8th Edition)	
M1B	4 (13%)
M1C	4 (13%)
M1D	22 (74%)
LDH level	
Normal	18 (60%)
Elevated	10 (33%)
Unknown	2 (7%)
Localisation of metastases	No. patients
Brain	22 (73%)
Lungs	4 (13%)
Epipharyngeal region	1 (3%)
Retrobulbar region	1 (3%)
Renal	1 (3%)
Adrenal gland	1 (3%)
Number of irradiated metastases	No. patients
Solitary (1)	19
Multiple (2 or more)	11
Median (range)	4 (1–5)
Size of metastases (cm)	
Median (range)	1.25 cm (1–4 cm)
Number of session of radiotherapy	No. patients
1	20 (67%)
2 or more	10 (33%)
Type of SRT	No. patients
Single-fraction SRS	18 (60%)
Fractionated SRT	12 (40%)
All radiation session no.	47 (100%)
Doses	
SRS	32 (68%)
3x20 Gy	1 (2%)
1x19 Gy	1 (2%)
1x18 Gy	16 (34%)
1x17 Gy	4 (9%)
1x16 Gy	8 (17%)
1x15 Gy	2 (4%)
Fractionated SRT	15 (32%)
8x7,5 Gy	1 (2%)
8x6,5 Gy	1 (2%)
5x12 Gy	1 (2%)
5x11 Gy	1 (2%)
5x9 Gy	1 (2%)
5x6 Gy	5 (10%)
5x5 Gy	2 (4%)
3x11 Gy	1 (2%)
3x8 Gy	1 (2%)
3x7 Gy	1 (2%)

20 patients (67%) were treated with a single session of stereotactic radiosurgery and 10 patients (33%) received 2 or more sessions for the target lesions. 16 patients (60%) were treated by single fraction SRT, and 14 patients (40%) received fractionated treatment.

The median number of metastases was 4 (range 1–5). All lesions were treated with linear accelerator (LINAC) based systems. 21 (70%) received SRS on CyberKnife, six patients (20%) on VitalBeam2, 3 (10%) patients on TrueBeam.

In the case of 10 patients (33%) SRS was the first line treatment, and we started systemic therapy afterwards. 20 patients (68%) were treated with SRS combined with systemic treatments. Eight patients (27%) received SRS combined with targeted Braf-MEK inhibitors, eight patients (27%) combined with immunotherapy, and four patients (14%) with chemotherapeutic agents (Table 2).

**TABLE 2:** Treatments.

Prior systemic therapy	
Targeted therapy (Braf-MEK inhibitor)	8 (27%)
Immunotherapy (PD-1, CTLA-4 inhibitor, combined immunotherapy)	8 (27%)
Chemotherapy	4 (13%)
No prior sytemic therapy	10 (33%)
Systemic treatment after the SRS	
Remained the same agent	16 (53%)
Changed	3 (10%)
No systemic treatment after SRS	4 (13%)
Started systemic therapy	7 (24%)

After the SRS in 16 cases, we could continue the original systemic treatment, in four cases we didn’t begin any systemic therapy but observation, and in only three cases we had to change to another therapeutic regime.

**FIGURE 1:**
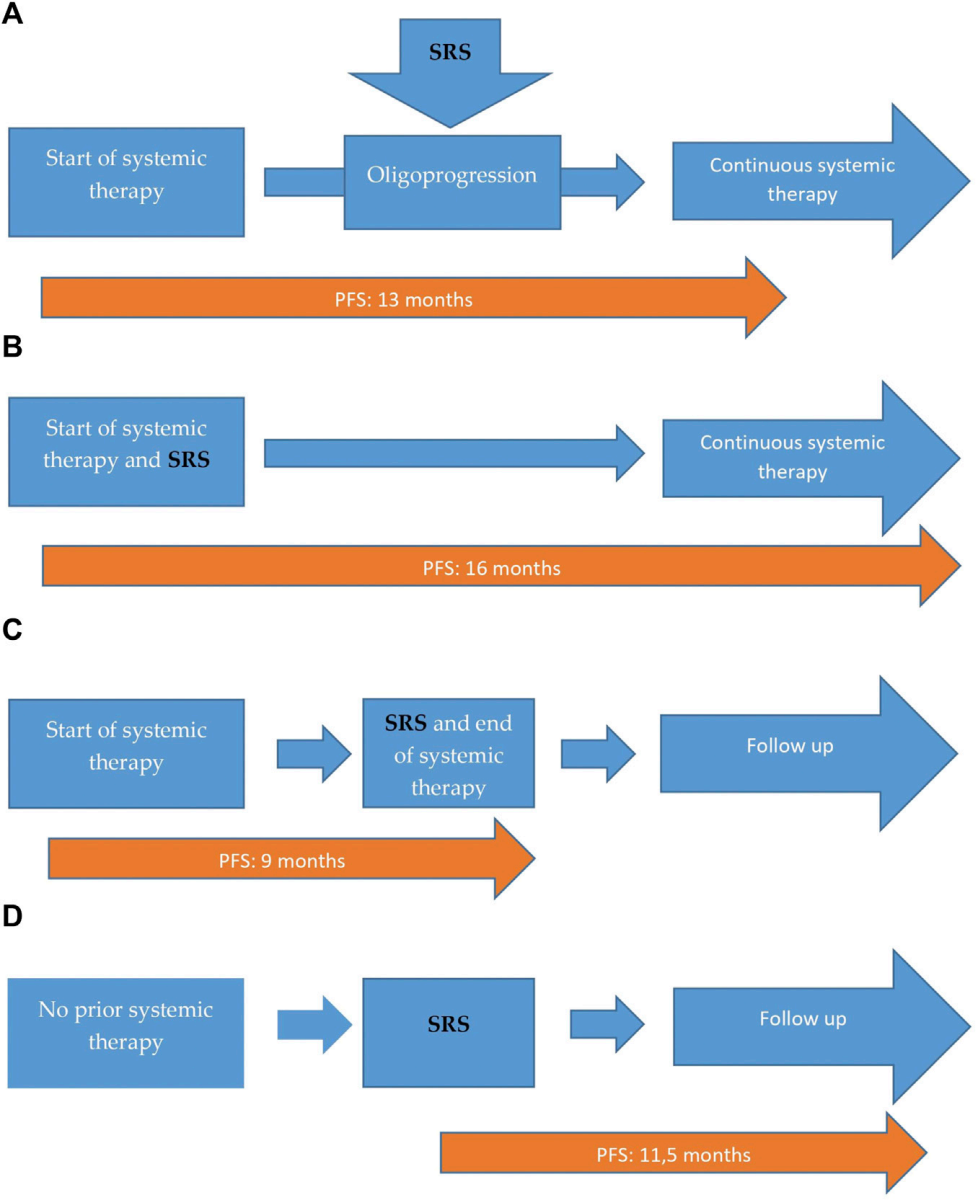
Distribution of our patients according to the therapeutic sequences. **(A)** Patients who received systemic treatment before the SRS. Patients no: 17 (57%), with 13 months of median PFS. **(B)** Patients who received SRS and systemic simultaneously. Patients no: 8 (27%), with 16 months of median PFS. **(C)** Patients who did not receive additional systemic therapy after SRS. Patients no: 3 (10%), with 9 months of median PFS. **(D)** Patients who received only SRS, with no prior or additional systemic treatment. Patients no: 2 (6%), with 11.5 months of median PFS.

Seventeen patients received systemic therapy before the SRS, eight patients received SRS and systemic treatment simultaneously, three patients did not receive additional systemic therapy after the SRS and two patients didn’t received systemic therapy before or after radiotherapy (Figure 1).

### Response and Survival Outcomes

The median follow-up was 20 months (range: 4–52), the minimum was 4 months. We achieved local response of the irradiated tumor: partial response (PR) in 14 patients (47%), complete response (CR) in six patients (20%), stabil disease (SD) in three patients (10%) and progressive disease (PD) in seven patients (23%). Local objective response rate (ORR) was achieved in 23 patients (77%)Regarding the systemic disease: PR in 12 patients (40%), CR in six patients (20%), SD in two patients (7%) and PD in 10 patients (33%); systemic ORR was detected in 20 patients (67%). At the time of the analysis (October 2020) fourteen patients (46%) were still undergoing treatment or observation, and sixteen patients (54%) deceased. We managed to reach 12.5 months PFS. 13 months of median PFS was observed in patients (no: 17, 57%) who received systemic treatments before the SRS (Figure 1A) and 16 months of median PFS was detected at patients (no: 8, 27%) who received SRS and systemic treatments simultaneously (Figure 1B). We reached 9 months of median PFS in patients (no: 3, 10%) who did not receive additional systemic therapy after the SRS (Figure 1C) and 11.5 months of median PFS in patients (no: 2, 6%) who received only SRS, with no prior or additional sytemic treatment (Figure 1D).

We reached median OS of 20 months from the beginning of systemic therapy or the time of SRS at patients we did not receive systemic therapy, before or after the radiotherapy.

**FIGURE 2:**
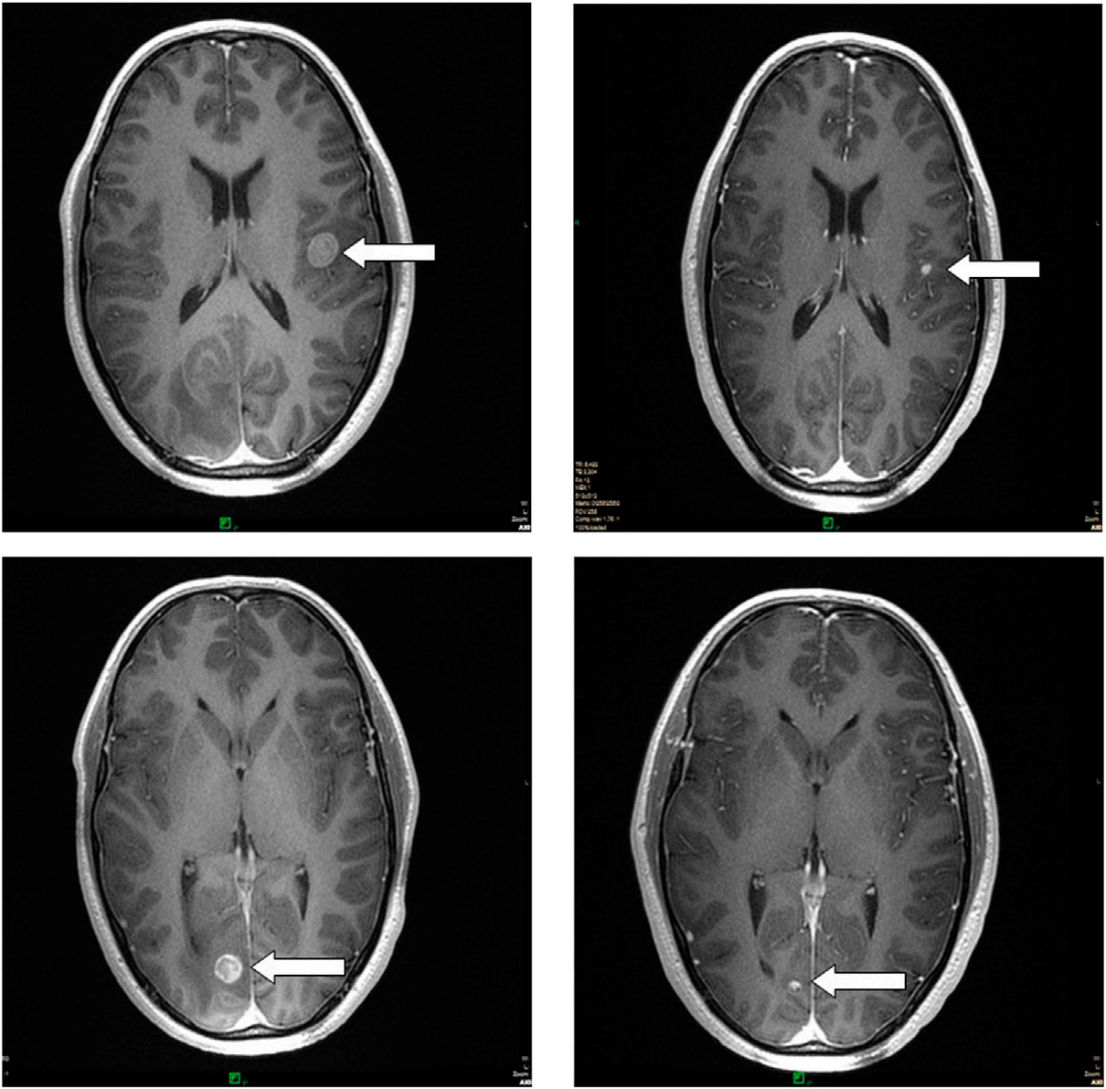
Follow-up MRI scans show complete regression of two brain metastases, 6 months after the radiosurgery, treated simultaneously. The patient received targeted therapy as systemic treatment.

**FIGURE 3:**
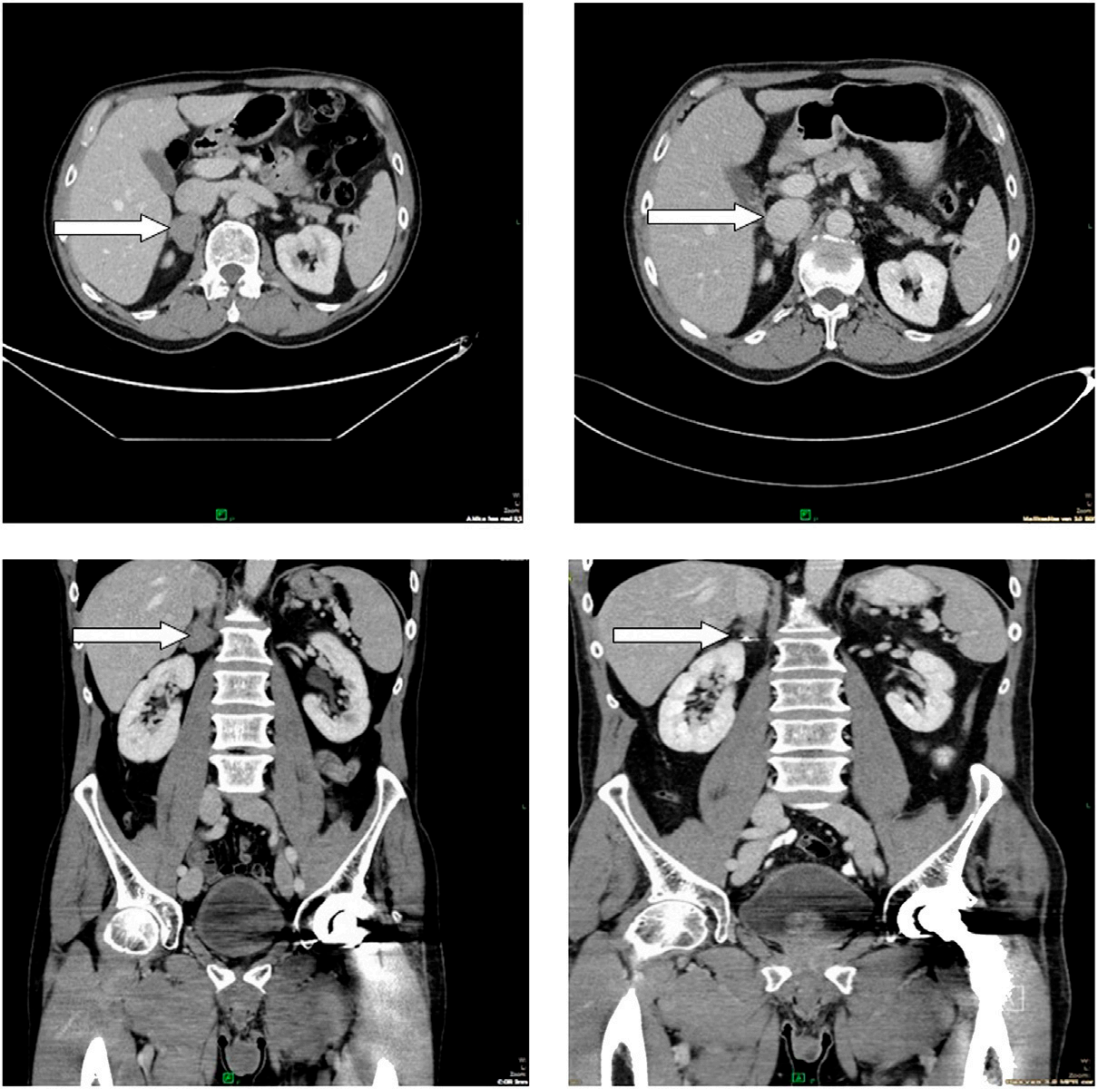
Follow-up CT scans show partial regression in the adrenal gland, 6 months after the radiotherapy. The patient received checkpoint inhibitor, as systemic therapy.

**FIGURE 4:**
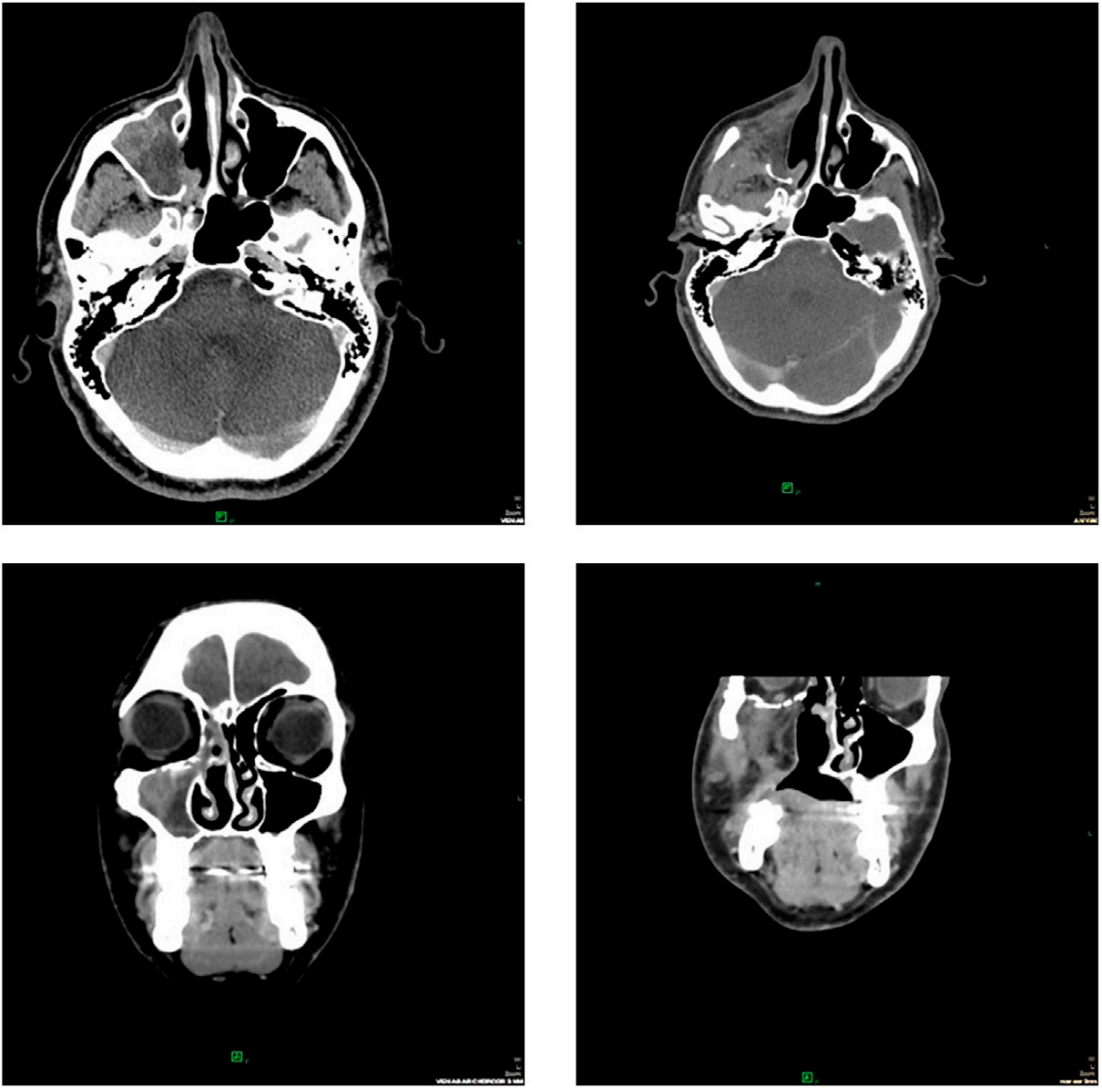
CT scans after 6 months of the irradiation showed partial response of the primary melanoma in the nasal cavity. The patient received chemotherapy as systemic treatment.

In Figures 2–4, follow-up CT scans of three patients, after 6 months of the irradiation shows significant regression of the brain (Figure 2), adrenal gland (Figure 3) and nasal cavity (Figure 4).

**FIGURE 5:**
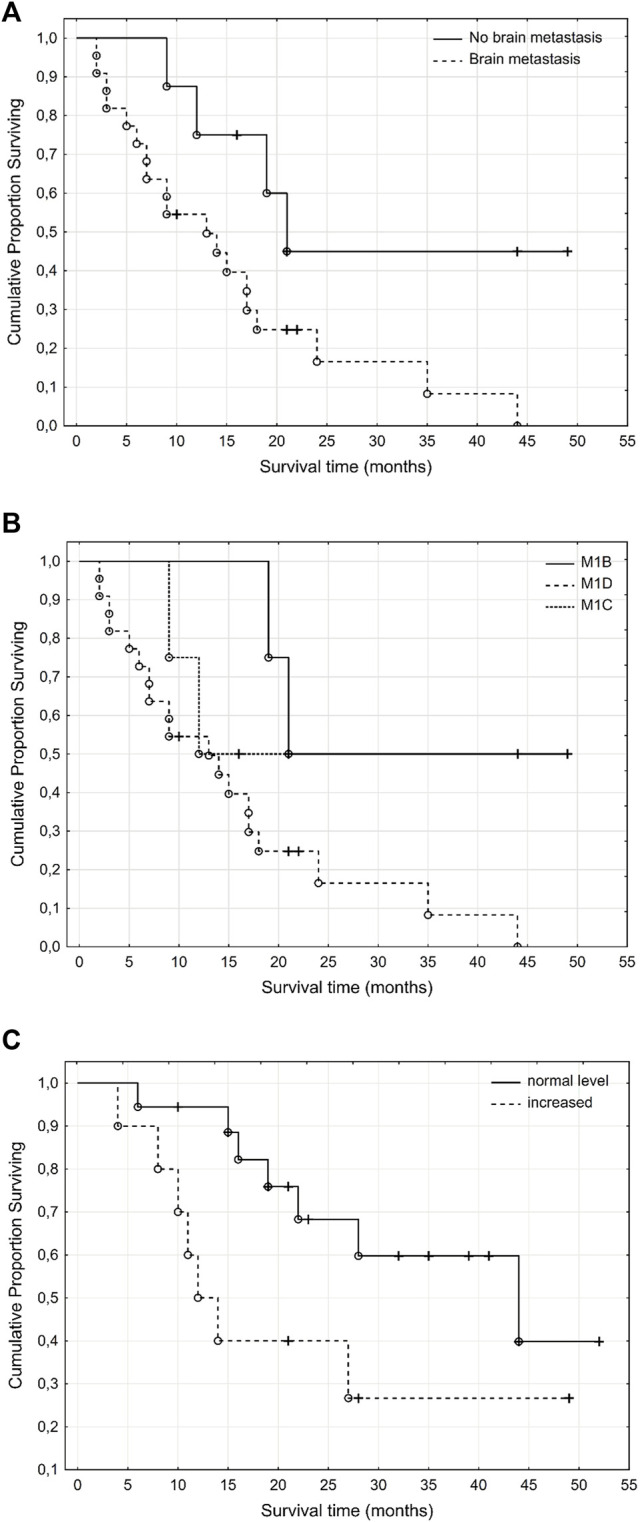
Kaplan Meier curves of melanoma patients according to presence of brain metastases **(A)**, M stage **(B)** and LDH levels **(C)** at the beginning of treatment. Presence of brain metastases significantly and the M stage at the beginning of the systemic therapy non-significantly impaired progression free survival while the elevated LDH levels in the blood at the beginning of the systemic therapy non-significantly impaired overall survival. **(A)**, PFS- brain metastases (*p* = 0.02). **(B)**, PFS-M stage (*p* = 0.084). **(C)** OS–LDH level in blood (*p* = 0.068).

In our study the Kaplan-Meier curves (Figure 5) showed that the presence of brain metastases (Figure 5A) significantly (*p* = 0.02) negatively impaired PFS and the patients M stage (Figure 5B) non-significantly (*p* = 0.084) impaired PFS regarding the therapeutic period from the beginning of systemic treatment. Survival data was based on the last visit, or the death of the patient. Starting LDH levels in the blood non-significantly (*p* = 0.068) impaired OS regarding the therapeutic period from the beginning of systemic treatment (Figure 5C).

### Therapy Related Adverse Events

Adverse events were recorded in a total of 27 events at 16 patients (53%). Nine patients (30%) received targeted therapy, three patients (10%) received immunotherapy and three patients (10%) received chemotherapy. One patient (3%) received no systemic therapy either before or after treatment.

Most of them were related to the brain (twenty cases, 74%), such as nausea or vomiting (six cases, 22%), headache (six cases, 22%), dizziness (six cases, 22%), aphasia (one case, 4%), and seizure (one case, 4%). In the case of one patient (4%),—who has received targeted therapy in addition to radiotherapy-hospitalization was imminent, due to the grade 3 epileptic seizure (Table 3). Dehydration therapy with high dose steroid (parenteral) and mannitol was applied, magnetic resonance imaging (MRI) scans showed no signs of radionecrosis. We concluded that the seizure was the result of radiotherapy-induced edema, and the symptoms completely disappeared 1 day after therapy.

**TABLE 3:** Therapy related adverse events.

Therapy related adverse events	Grade 1 No (%)	Grade 2 No (%)	Grade 3 No (%)
Number of patients with at least one adverse event	11 (37%)	4 (13%)	1 (3%)
All brain related AE	13 (43%)	6 (20%)	1 (3%)
Nausea or vomiting	5 (17%)	1 (3%)	0
Aphasia	1 (3%)	0	0
Dizziness	4 (13%)	2 (6%)	0
Headache	3 (10%)	3 (10%)	0
Seizure	0	0	1
Non brain related AE	6	1	0
Eyesore	1 (3%)	0	0
Esophageal irritation	1 (3%)	0	0
Coughing	2 (6%)	0	0
Pain in the radiated organ	1 (3%)	0	0
Interstitial pneumonitis	0	1 (3%)	0
Sweating	1 (3%)	0	0

We observed non-brain related adverse events after the radiation therapy of visceral metastases in seven cases (26%).

Non-brain related adverse events were coughing (two case, 7%), eyesore (one case, 4%), sweating (one case, 4%), pain (one case, 4%), esophageal irritation (one case, 4%) and interstitial pneumonitis (one case, 4%). All of them were grade 1 events, except for one case of pneumonitis. It was evaluated as grade 2. It was observed only on computer tomography (CT) images and the patient did not have any clinical symptoms; he received oral intermediate dose of methylprednisolone for a month in a constantly decreased dose, completed with oral antibiotics (amoxicillin) for 2 weeks. One month after treatment, CT examinations showed regression of the pneumonitis.

## Limitations

The limitations of our study include the small number (30) of patients, most of our eligible patients (no: 22, 73%) had brain metastases and the study’s retrospective nature.

## Discussion

According to our results, the effectiveness of systemic therapy may be increased, if it is combined with stereotactic radiation, in case of oligoprogression/oligorecurrence in metastatic melanoma. The treatment of advanced cutaneous melanoma changed radically, since 2011. New therapeutic modalities, such as the immuno- and targeted therapies resulted in statistically significant prolonged PFS and OS in patients with metastatic melanoma (11). However, in many instances, the systemic therapeutic modalities alone did not prove sufficient. In cases of oligoprogression, systemic therapeutic modalities can be completed with local treatment options such as metastasectomy, stereotactic radiotherapy, electrochemotherapy, radiofrequency tumor ablation (RFTA) (12). The main direction in the treatment of patients with advanced melanoma is systemic therapy, but in specially selected cases, such as the presence of oligometastases or oligoprogression of the disease, local therapeutic modalities can be used succesfully. The definition of oligoprogression varies between different neoplasms, but it is mainly defined as a clinical state, in which the progression is at a limited degree, meaning it affects less than three regions, and one or two metastases, while the systemic therapy controls the disease on all the other sites. Approximately 4%–10% of melanoma patients treated with immune- or target therapy develop oligoprogressive disease (13). This type of progression is related to acquired resistance, and biologically different from generalized progression, which is caused by pre-existing or secondary therapeutic resistance (14). Due to this specific attribute, oligoprogressive disease can be treated efficiently with various local therapies, and thereby acquired resistance can be controlled, and we are allowed to continue the previously started, effective systemic therapy. Brain metastases are common in melanoma compared to other malignancies, which may explain why 22 (74%) of our patients were treated for cerebral metastases, but the tumor can often metastasize other peripheral regions (6,15,16). The result of several retrospective analyses concluded that some patients treated with checkpoint inhibitors who developed progression had benefited from continuing the treatment. In contrast, targeted therapy is rarely continued alone after progression without local treatment. There are certain trials, such as COMBI-d (NCT01584648) and COMBI-v (NCT01597908) in which most of the patients were treated with local therapies (surgical, radiotherapy, about 50% in both trials) besides the targeted therapy (5). While in case of the extremely painful osseous metastases and the multiplex cerebral metastases of melanoma when the treatment is mostly palliative or whole brain radiation therapy (WBRT), in the presence of solitary- or oligometastases (<5) we can achieve the complete remission of the metastases with local radiotherapy (17,18). In our study CR was achieved at six patients (20%). Our possibilities are improved by the presence of modern stereotactic radiosurgery since the higher doses have much more biological effect than the conventional radiotherapeutic choices have. A rare phenomenon has been known for decades, when clinicians detected antitumor effects in localisations far from the originally irradiated lesion. This rare phenomenon was called an abscopal effect. By recent experiences, this irradiation triggered abscopal effect depends on the immune system of the patient. If this assumption is proven, then combined radio-immunotherapy available to us as an effective way to defeat the immune escape mechanisms of various malignant neoplasms, such as metastatic melanoma, and it can possibly increase therapeutic response. Ionizing radiation also can increase the vigilance of T-cells against malignant cells, as shown by preclinical studies. According to another theory, the abscopal effect is activated by neoantigens released from the previously irradiated and thereby destroyed malignant cells (19–21). However in 30 patients, we cannot clearly state, that abscopal effect occurred, but we assumed that it contributed to our good results. In contrast, the radiosenzitizing effect of targeted therapies can cause severe side effects, if applied concomitantly with radiotherapy, such as abscess in the brain, or severe, potentially fatal rectal fistula. According to international protocols we suspended targeted therapies 1 day before and after SRT/SRS which proved to be safe, considering that no serious adverse-events were observed (22). In our study the Kaplan-Meier method and log-rank statistics showed significance between the presence of brain metastases and progression-free survival; and near significance between the M stage and overall survival, and the LDH level was detected (Figure 2). In various clinical trials investigating the efficacy of systemic therapies in melanoma, the median PFS was between 6 and 12 months (23–26). In our study 12.5 months of PFS and 20 months of median OS was achieved with continuous systemic therapy, combined with SRT/SRS. Ratnayake et al. (27), published the results of a phase one clinical trial in which their purpose was to determine the maximum tolerated dose of stereotactic radiation therapy, combined with immunotherapy. Their study also examined the effects of timing, and dosing of stereotactic radiotherapy and radiosurgery on the efficacy of the systemic treatment. They treated 24 melanoma patients with stereotaxic irradiation, combined with immunotherapy. They reached 2.2 months median PFS, and the median OS was 16.9 months, despite of them having examined patients without metastases in the central nervous system (CNS), and having treated patients who only received checkpoint-inhibitors as systemic therapy. In their study, they described numerous side effects, after stereotactic radiotherapy and radiosurgery. They maximized the dose of the SBRT at 15 Gy and their conclusion was that SBRT at 10 GY can be safely combined with checkpoint inhibitors. However, in our Institute the maximum dosage of radiotherapy was 20 Gy, most of our patients (*n* = 20, 67%) received stereotactic radiotherapy and radiosurgery with systemic therapy in addition, no serious side effects were observed. Besides the checkpoint inhibitors, there were patients in our study who received targeted therapy or chemotherapy as well. Feng et al. (28), published a study in which they treated 87 melanoma patients with stereotactic radiosurgery. The median survival was 6 months. Their results are worse compared to ours, but it is important to note that they only irradiated metastases localized in the CNS, however, seven patients of theirs had metastases in other sites, without SRT.

## Conclusion

In our study we found that systemic therapy combined with stereotactic radiation therapy may improve the efficacy of the treatment in advanced melanoma patients. Adverse events were tolerable. Larger numbers of cases are required to determine the exact indication in advanced melanoma.

## Data Availability

The raw data supporting the conclusion of this article will be made available by the authors, without undue reservation.

## Abbreviations

CNS, central nervous system; CPI, checpoint inhibitors; CR, complete response; CT, computer tomography; CTCAE, common terminology criteria for adverse events; ECOG, Eastern Cooperative Oncology Group; iRECIST, immune response evaluation criteria in solid tumors; LDH, lactate dehydrogenase; LINAC, linear accelerator; MRI, magnetic resonance imaging; ORR, objective response rate; OS, overall survival; PD, progressive disease; PFS, progression free survival; PR, partial response; RECIST, response evaluation criteria in solid tumors; RFTA, radiofrequency tumor ablation; SBRT, stereotactic body radiotherapy; SD, stable disease; SRS, stereotactic radiosurgery; SRT, stereotactic radiotherapy; WBRT, whole brain radiation therapy.
